# Editorial: One Health: The Parameters of an Eco-Sustainable Farm

**DOI:** 10.3389/fvets.2021.681288

**Published:** 2021-04-27

**Authors:** Mario Baratta, Gianfranco Gabai, Pietro Celi

**Affiliations:** ^1^Department of Veterinary Science, University of Turin, Turin, Italy; ^2^Department of Comparative Biomedicine and Food Science, University of Padua, Padua, Italy; ^3^Melbourne School of Land and Environment, The University of Melbourne, Melbourne, VIC, Australia

**Keywords:** sustainable agriculture, animal welfare, biodiversity, life cycle assessment, health biomarkers

The production of food of animal origin significantly contributes to GHG emission, land use, climate changes, and biodiversity loss. At the same time, in many areas around the world animal production is a crucial sector of human activities. The understanding of the interactions between livestock farming and environment and the development of measures to mitigate the carbon footprint of animal production systems, is gaining growing importance in the international decision makers' policy agenda. In addition, the consumers' awareness about quality and sustainability of the production cycle of animal food products, and the interest for setting quality brands associated with animal production are increasing. Consumers increasingly demand for food associated with sustainable production systems that contribute to the conservation of biodiversity, while confronting climate change by controlling GHG emissions, etc. The EU great diversity of agricultural systems associated with semi-natural habitats and rural landscapes of recognized environmental value is also essential for other productive systems, such as tourism.

For these reasons, the sustainability of diverse animal production systems should be assessed by a multidisciplinary approach focusing on the ecological and carbon footprint, animal nutrition, health and welfare, and the nutrition value of meat and dairy products.

This approach is even more important within the “One Health” context, where human, animal and environmental health are deeply interconnected. One Health is a global concept that involves all living things on the planet and that is interlinked by a common goal of health, sustainability and safety. The contribution of the veterinary sciences to this multidisciplinary approach is multi-faceted. However, in this Special Issue the focus has been placed on two main topics: (i) the control of animal health through new approaches given by precision livestock farming (biosensors), and (ii) the development and validation of biomarkers capable to identify potential harmful health issue in animals at an early stage. We are well aware that the control of animal health, specific to veterinary sciences, needs to be integrated to accommodate the various requests that the different subjects of the chain encounter.

Breeders, farmers, consumers, scholars of energy reserves, and the environmental impact are all part of this chain.

In this collection, four papers deal with the use of biomarkers to assess the conditions of health and welfare of farmed animals. Two reviews explore the current knowledge about the use of miRNAs as biomarkers of health and welfare in ruminants, pigs and poultry and the potential use of DHEA for the definition of stress in farmed animals. One original study investigates metabolite fingerprints in the urine to identify potential metabolite alterations and observes multiple urinary metabolite alterations, which are promising biomarkers to identify ketotic cows. Promising preliminary data about the use of bone markers for predicting milk fever in dairy cows are also presented. Animal welfare can be monitored by behavior recording using a livestock precision farming approach, and one study reports on how lying time is a variable related to animal adaptation and indicative of animal welfare. Subfertility is still a significant challenge for the sustainability of the dairy industry limiting herd productivity. The farmers and veterinarians' perception of this issue has been investigated and data suggest that accurate monitoring of reproductive status and early detection of fertility issues in individual cows is considered as essential. Finally, the increasing concern in society regarding the consumption of products of animal origin has drawn attention to the need of understanding how the production process could be carried out in a sustainable manner. In this collection, two studies report on holistic approaches to analyze environmental, economic, and social aspects. Data suggest that the use of local cow breeds maximize the efficiency in the use of territory resources, such as grasslands in a mountain environment, allowed dairy production to reduce emissions. In the case of lamb meat production, extensive traditional sheep farming has positive effects on local ecosystems and on animal welfare without ignoring the environmental advantages of intensification.

The collection of articles that we present is an attempt to start considering common paths for the different aspects that fall within the concept of eco-sustainability of an animal supply chain where interdisciplinary must become the ordinary way to study problems and the different points of view must be necessarily shared and evaluated. What emerges from the research reported here and from those that are increasingly published is that it is necessary to improve the ability to analyze and contextualize the supply chain in order to highlight its characteristics and eco-sustainability. The future that awaits research applied to animal production is that of greater integration between agriculture, animal husbandry, and the territory for a more effective conservation of resources and reduction of the impact of production, which is therefore increasingly requested by consumers.

## Author Contributions

All authors contributed to the elaboration and critical discussion of the Editorial.

## Conflict of Interest

The authors declare that the research was conducted in the absence of any commercial or financial relationships that could be construed as a potential conflict of interest.

